# International experiences of systems approaches: re-thinking policies and governance to transform agrifood systems

**DOI:** 10.1098/rstb.2024.0159

**Published:** 2025-09-18

**Authors:** Laura De Matteis, Dalia Mattioni, Pablo Garcia Campos, Elena Teodora Ilie, Esther Wiegers, Corinna Hawkes

**Affiliations:** ^1^ Agrifood Systems and Food Safety Division, Food and Agriculture Organization of the United Nations, 00153 Rome, Italy; ^2^ Department of Food, Agriculture and the Environment, University of Pisa, 56124 Pisa, Italy

**Keywords:** agrifood systems, food systems transformation, systems approach, systems thinking, governance, systems leadership

## Abstract

A profound transformation of purpose means that agrifood systems are expected to attain multiple sustainability outcomes, beyond producing enough food, towards achieving human and planetary health for current and future generations. Yet, despite the existing range of policies, innovations and interventions, agrifood systems transformation is hindered by short-term thinking, siloed approaches, power imbalances and linear mindsets. Persistent challenges demand a transformation of *how* action is taken. In response, people and institutions across the world are beginning to adopt different ways of working. Drawing inspiration from various countries, this article illustrates the promise and practice of delivering agrifood systems transformation through a *systems approach*. Key insights confirm that first, systems transformation requires long-term programmatic and investment cycles that leverage the interconnectedness of the agrifood system. Second, effective transformation needs to value the role of systems leaders to catalyse the change process, while also enabling inclusive governance processes that empower a diversity of voices to participate in decision-making. Finally, tangible outputs (e.g. change in policy or governance body or investment) and intangible outputs (e.g. change in thinking, relationships, connections and agency) of a systems approach are observed. Future agrifood systems interventions should promote both types of outputs, as essential components of transformation.

This article is part of the theme issue ‘Transforming terrestrial food systems for human and planetary health’.

## 1. Introduction: the opportunity for transformative action

Acting to transform agrifood systems^1^ has never held such promise. In recent years, an extensive body of evidence and experience has revealed numerous opportunities to transform agrifood systems to address some of the world’s most pressing challenges [2,3]. In 2021, the United Nations Food Systems Summit (UNFSS) aimed to highlight this opportunity by fostering collective commitment of a broad range of stakeholders towards realizing a resilient, equitable and sustainable global food system, in line with the objectives set forth in the Agenda 2030. A total of 126 United Nations (UN) member states and cities from across the globe responded to this call for action, convening multi-stakeholder dialogues led by government-appointed National Convenors. This process informed the formulation of multi-sectoral strategies envisioning the agrifood systems of the future, the so-called ‘UNFSS national pathways for food systems transformation’ [4,5].

These developments show that expectations of national and sub-national governments and the global agrifood systems community have changed: rather than being seen only as a provider of sufficient food and agricultural products, agrifood systems are now perceived as essential contributors to inclusive economic growth, environmental protection, healthy diets and nutrition, and gender equality [6]. This is why they were identified as one of the essential six transitions towards the achievement of the Agenda 2030 for Sustainable Development, the SDGs [7]. This marks a profound transformation of purpose for agrifood systems.

To this end, an extensive range of policies, institutional reforms, innovations and investments are being delivered in agrifood systems. For example, voluntary progress reports submitted by 101 countries in preparation for the UNFSS +2 (which was held in 2023 to take stock of the progress since the 2021 UNFSS) documented that 70% of the reporting countries, particularly low- and lower middle-income ones, have established or strengthened food systems governance to facilitate cross-sector collaboration and engage multiple stakeholders at various levels. In addition, 67% of the reporting countries, especially middle- and high-income nations, deliberately integrated the priorities outlined in their National Pathways into their overarching national development plans and strategies, committed to ensure policy alignment and coherence [4].

However, these actions are not yet adding up to transform agrifood systems, i.e. fundamentally change the way agrifood systems function towards a better set of outcomes on multiple fronts [8,9]. Addressing today’s complex interactions between food production, environmental sustainability and human health is still hindered by short-term thinking, siloed and sectoral approaches, power asymmetries among agrifood systems actors, as well as linear mindsets [2].

The transformation of purpose has thus not yet translated into a transformation of outcomes. By any measure, the food insecurity, malnutrition and ill-health associated with agrifood systems are unacceptably high; agrifood systems are one of the major drivers for climate change, biodiversity loss and environmental degradation; and economic development in agrifood systems is insufficiently inclusive, perpetuating deep inequalities [1]. Agrifood systems are also themselves changing as a result of conflict, climate-related disasters, economic instability, urbanization and new technologies [10].

In response, many people and institutions across various countries are beginning to embark on a longer-term journey to adopt different ways of working through a systems approach, recognizing that persistent challenges affecting agrifood systems demand a transformation of how action is taken through a process of profound and systemic change. Countries are gradually departing from linear and siloed ways of thinking and working with the intent to steer ongoing agrifood systems transformations towards greater social, economic and environmental sustainability [11].

Drawing inspiration from positive examples from selected countries, this article illustrates how stakeholders from different parts of the world are working to transform their agrifood systems in ways that exemplify some key elements of a systems approach: the capacity to think systemically and shift mindsets, beliefs and assumptions [9]; implementing policies that recognize and leverage the interconnectedness of different components and outcomes of agrifood systems and inter-related systems through mutually reinforcing actions [8]; valuing knowledge systems that transcend narrow analytical siloes; strengthening collaborative relationships between a diversity of people and institutions [12] through participatory and inclusive governance; allocating budgets to incentivize cross-sector planning and complementary actions; and establishing new approaches for monitoring, evaluation and learning (MEL) mechanisms that allow experimentation, adaptation and critical reflection [13]. The cases also exhibit the competencies of systems leadership, the set of skills and capacities needed to catalyse, enable and support the process of systems-level change [14] with an explicit intent to ‘steward’ processes of systemic and transformative change that include changes in relationships and structures and their underlying mindsets and values [15].

Some of the examples are drawn from activities undertaken in the context of the UNFSS. Under the leadership of its UNFSS National Convenor, Morocco fostered interaction among multiple sectors through the collective formulation of a national policy roadmap, the establishment of a food systems governance committee and the initiation of an integrated, multi-sectoral investment portfolio; these advancements are instrumental to align actions to transform Morocco’s food system towards food and water security and equitable growth. Still in the framework of the UNFSS, Albania formulated a collective vision to revitalize rural economies through sustainable agritourism. This was grounded in territorial reality, involving municipalities, farmers, entrepreneurs and vocational training institutes to participate in inclusive policy dialogues. Additionally, the Summit stimulated Rwanda to introduce cross-sectoral policy innovation in its latest Strategic Plans for Agricultural Transformation and launch a policy learning programme aimed at equipping public institutions with systems thinking skills. Although not directly triggered by the process set in motion under the UNFSS, the article also examines the cases of Costa Rica and the Pacific islands. In Costa Rica, an integrated portfolio of actions enabled the trade-off between coffee productivity and environmental welfare to be tackled by fostering a public–private partnership aimed at keeping coffee production levels high while mitigating its environmental impact. In the Pacific islands, faced with one of the highest levels of obesity in the world, a series of different actions, ranging from science–policy interface to consumer engagement, were taken to lay the foundations for more resilient Pacific islands food systems delivering healthy diets for all.

Though from very different contexts, all the cases presented can provide inspiration for the UK and beyond.

## 2. Methodology

The authors used select case studies drawn from Food and Agriculture Organization of the United Nations (FAO) projects, conversations with key informants, and observations throughout the projects’ implementation to collect data for the illustration of how elements of a systems approach were adopted and what change process they stimulated. The criteria used to select the case studies were the extent to which the projects aimed at embracing a systems approach and the amount of documentation that could allow reconstruction of the use of a systems approach. The authors also aimed to reach a certain geographical balance to account for sociocultural and economic differences across regions.

## 3. Morocco: transforming governance of agrifood systems

In 2021, as part of their engagement in the UNFSS process, the government of Morocco began to advance a more systems-based approach to address compounded agrifood challenges.

This engagement with food systems followed several years of policies and investments designed by Morocco’s Ministry of Agriculture to ‘green’ agrifood production and advance socio-economic development in rural areas, as exemplified by Plan Maroc Vert in 2008 and Generation Green in 2020. These strategies were conceived in response to climate-driven variations and chronic water stress (around 600 m^3^ of water per capita per year, lower than the stress threshold of 1000 m^3^), which have made Morocco’s contribution of agriculture to gross domestic product (GDP) volatile, given its heavy reliance on irrigation, owing to erratic rainfall patterns [16,17]. At the same time, steady demographic growth, predominantly rural poverty [18] and consecutive years of drought have contributed to keep the debate around food and water security high on the political agenda in recent years [19–21].

Stimulated by the momentum generated by the UNFSS process and a commitment to move beyond a siloed approach to accelerate multiple development objectives, the Moroccan Ministry of Agriculture, under the leadership of the UNFSS National Convenor, demonstrated systems thinking to advance equitable, resilient and sustainable food systems. Recognizing that a systems transformation requires commitment and coordinated action of multiple sectors, the Ministry convened two rounds of multi-stakeholder policy dialogues between 2021 and 2023 with the support of FAO’s Sustainable Food Systems in the Mediterranean (SFS-MED) initiative. These dialogues fostered open and constructive engagement among sectors, enabling Morocco to build consensus around a shared, integrated agrifood systems agenda. For the first time, multiple national agencies were involved in the formulation of a national policy roadmap for agrifood systems transformation that articulates cross-sectoral objectives, including green and equitable economic growth empowering women and youth, environmental sustainability and health and social protection [22]. This roadmap is a concrete outcome of this coordinated effort, as it consolidates and aligns the priorities and objectives of a series of national strategies under one umbrella (including Generation Green, the National Nutrition Strategy, and the National Strategy for Waste Reduction and Valorization), reflecting a shift towards mutually reinforcing policies across sectors.

In 2024, Morocco took a major step toward institutionalizing cross-sectoral governance to implement the priorities identified collectively, by launching the National Committee for Food Systems Transformation (CNTSA) [23]. This committee is responsible for providing shared strategic direction, coordinating efforts, monitoring the implementation of the national roadmap, and identifying investment opportunities to drive forward the agrifood systems transformation agenda. The UNFSS Convenor dedicated time to build trust and strengthen relationships with other line ministries through bilateral and multilateral negotiations. As a result, in another first, four ministries now constitute the CNTSA core group, marking a formal commitment to cross-sectoral leadership and joint responsibility: (i) the Ministry of Agriculture, Maritime Fisheries, Rural Development and Water and Forests; (ii) the Ministry of Industry and Trade; (iii) the Ministry of Health and Social Protection; and (iv) the Ministry of Energy Transition and Sustainable Development. In addition, the scientific community, civil society and private sector compose the CNTSA ecosystem of support, by participating in national consultations and the operation of the technical sub-committees.

Finally, this expanded focus on multi-sectoral priorities and multi-stakeholder governance has already begun to catalyse more integrated investments. Influenced by its engagement in the policy dialogues convened by the government and its role as co-chair of the Development Partner Group on food security together with the FAO, the World Bank designed a new portfolio of investments that spans across all areas of the national food systems roadmap. This constitutes a significant departure from the siloed approaches of the past, shifting away from narrowly focused investment programmes—often on water security—towards incentivizing complementary actions [8].

## 4. Albania: building a shared vision

In 2021, the UNFSS stimulated Albania’s government to elaborate a national pathway for food systems through a series of dialogues convened by the Ministry of Agriculture and Rural Development (MARD), with the participation of central and local stakeholders. The pathway focused on a priority problem faced by Albania: unequal and unsustainable economic growth. Although agriculture makes a significant contribution to the national GDP (19%) and total employment rate (34%), the majority of Albania’s economic output is generated by its cities, whose rapid expansion is threatening natural resources and increasing vulnerability to natural disasters [11,24]. Inadequate rural infrastructure and the depopulation of rural areas due to significant outmigration, particularly of young women and men, has contributed to widen the rural–urban gap. To this, we must add an ageing farm population that faces challenges to meet food safety standards (including owing to low levels of phytosanitary controls) and new consumer demands. Conversely to these trends, incoming tourist flows more than doubled in under a decade, thanks to the attractiveness of Albania’s landscapes, wealth of biodiversity and a rich culinary tradition. To confirm this heightened attention towards biodiversity and natural resources, Albania established in 2023 the Vjosa Wild River National Park, protected by the International Union for Conservation of Nature [11].

In this context, the national pathway laid out a vision of shared rural–urban prosperity through food systems that drive sustained, inclusive and sustainable economic growth. The pathway also identified specific entry points to transformative change. This included agritourism and short value chains as mechanisms of development. By applying systems thinking, participants in the development of the pathway were able to see agritourism as a leverage point with catalytic potential for multiple interconnected outcomes, including entrepreneurial diversification in rural areas, environmentally friendly agriculture, and local biodiversity. The transformative potential of agritourism also comes from its potential to build short food supply chains that strengthen new relationships between multiple sectors, including agriculture, food processing and retail, tourism, environment and cultural heritage. At the same time, agritourism bridges the gap between rural producers and urban consumers, influencing societal perceptions and values about agriculture, food and sustainability, while building trust and respect along the agrifood chain [25].

The next step to advance the development of agritourism in the country was to intentionally design a process to foster shared directionality across the different stakeholders in different parts of the system, recognizing they brought different positions, perspectives and interests. The method used by the MARD and FAO, in the framework of the SFS-MED initiative, was to convene local dialogues in three districts and decentralize the discourse around agrifood systems transformation. By bringing together agritourism entrepreneurs, farmers, micro-finance institutions, municipality representatives, extension service providers and vocational training institutes, including culinary schools, the intent was to valorize other forms of knowledge closer to the realities on the ground.

These dialogues fostered systems thinking and collective learning by creating a safe, inclusive and publicly recognized space where people felt heard and empowered to engage in meaningful, cross-sectoral exchange. By facilitating interactions among actors from different sectors, the process allowed them to navigate differences, collectively reflect and learn from one another, and design a portfolio of complementary and mutually reinforcing actions. This way, new realizations emerged from these dialogues, such as, for example, the need for enhanced collaboration between the education system, agrifood businesses and rural communities to equip youth with the skills required to become the agrifood innovators of the future—an approach aimed at counteracting rural depopulation. Another realization concerns the need to include public health perspectives in agrifood systems dialogues, given the importance of increasing the coverage of healthcare centres in rural areas to allow easier access for farmers, agritourism operators and tourists [23]. To promote accountability and strengthen agency from local to national levels, the resulting collective vision to revitalize rural economies and protect natural resources and agrobiodiversity through sustainable agritourism was presented at a national consultation. This roundtable was co-chaired by the UNFSS National Convenor (Deputy Minister of Agriculture and Rural Development), the UN Resident Coordinator and the FAO and was attended by the Ministry of Tourism and Environment, public health agencies, academia, agrifood business operators, civil society and UN agencies [11].

The result of this process was to provide a foundation for the planning and implementation of more cohesive, cross-sectoral interventions. The establishment in 2023 of an agritourism department within MARD tasked with liaising with the Ministry of Tourism and Environment to implement joint actions is a tangible output.

## 5. Costa Rica: taking action for long-term sustainability

Since the 1980s, the government of Costa Rica, together with agrifood system stakeholders, has invested in agrifood exports to strengthen the country’s position within global markets. However, the Rio Earth Summit in 1992 sparked growing concern about the environmental sustainability of this export-oriented agrifood system model. A ‘sense of urgency’ emerged as data revealed increasing degradation of soil and water related to the intensification of export crop production. This raised at the time, and continues to raise, concerns for both environmental and economic sustainability, given that 17% of Costa Rica’s population of 5 million citizens depend on agriculture for employment [26].

The shared urgency prompted the government to incorporate sustainability ambitions into its national (food system) policies. These efforts include long-standing interventions in the coffee sector since the 1990s, the 2011−2021 Food and Nutrition Security Policy and the National Policy on Sustainable Production and Consumption 2018−2030. Most recently, in 2022, Costa Rica developed its National Pathway for Sustainable Food Systems as part of the country’s engagement in the UNFSS process [26,27].

The process of taking a more holistic perspective to agrifood systems is exemplified here through a focus on interventions in the coffee sub-system in the 1990s. At the end of the 1980s, coffee was one of the Costa Rica’s main exports, but with decreasing world prices and growing environmental disruptions, the government and other key actors have been taking steps to enhance the sustainability of the sector while keeping it competitive through sustainability certification schemes. The pathway to enhancing sustainability embraced systems ways of thinking and working. First, collective working by key institutions such as the Coffee Institute of Costa Rica (ICAFE) and the Ministry of Agriculture showed considerable political will to steer the sub-sector into the direction of greater environmental sustainability and ‘quality’ while maintaining its economic sustainability. The ICAFE is a multi-stakeholder institution composed of public and non-public actors, such as private companies, and large and small cooperatives. This inclusive governance mechanism strives to bring together different people and institutions across the coffee sector, as a foundation to advocate for a multidimensional transformation towards more sustainable production, driving innovation to maintain high coffee quality while also ensuring fair profits for all actors.

Second, complementary investments and actions were designed to build on and strengthen one another. The strategy taken by ICAFE was indeed to design a portfolio of public and private investments across the coffee subsystem with the specific intention of developing the economic and environmental sustainability of the sector. This included enhancing knowledge of the environmentally sustainable agronomic practices through direct economic engagement of public sector research and development (R&D) in agro-forestry techniques. It also included payment for environmental services and the creation of Nationally Appropriate Mitigation Actions (NAMAs)—the first to be set up globally—to create alliances with and leverage private investments [28]. The Coffee NAMA created a sustainability certificate on price premiums as an incentive for the private sector to invest in sustainable coffee, and implemented climate change mitigation actions at more than 60 coffee mills [28]. This portfolio of actions enabled the coffee sub-sector to achieve both economic growth and significant environmental improvements. Coffee is now Costa Rica’s third largest export crop, while its carbon footprint has strongly declined compared with previous decades.

While the coffee sub-sector was not initially selected as a strategic entry point for broader food system transformation, it did result, nonetheless, in inspiring and encouraging the emergence of other initiatives aimed at achieving environmental, economical and societal co-benefits, such as the Livestock NAMA. These past and ongoing efforts illustrate the transformative potential of making connections between different people and institutions across the system and coordinating actions towards a common direction. In the case of coffee, it is the portfolio of complementary actions related to knowledge, certification and public economic incentives that led to the success of the initiative. Such examples illustrate how coherent, cross-sectoral strategies can drive systemic change and may serve to encourage a wider societal debate over agrifood systems transformation, informing the implementation of UNFSS food systems pathways.

## 6. Pacific islands: co-creating knowledge to build the foundations of change

The incidence of non-communicable diseases (NCDs) in the Pacific island countries of Fiji, the Solomon Islands and Vanuatu is among the highest in the world. In Fiji, the most populous of the three, with only 900 000 residents, obesity affects one in three adults, particularly low-income women living in urban areas. Shifts towards unhealthy diets in the region are the result of compounded factors, including food prices and availability, dependency on imported foods and agricultural inputs, and vulnerability to climate-related loss of land [11].

To respond to these interconnected challenges, between 2016 and 2022, the FAO and the European Union, through the Food and Nutrition Security Impact, Resilience, Sustainability and Transformation (FIRST) Programme, promoted the adoption of a systems approach to policymaking and agrifood-related interventions in the region. As part of a whole-of-society engagement in agrifood systems transformation, the FAO worked with the Consumer Council of Fiji to foster the participation of universities, consumer groups and the media. This initiative aimed to promote systems thinking among consumers by facilitating understanding of the interdependent factors that influence diets and NCDs. This materialized in a partnership with the *Pacific Island Food Revolution* (PIFR), a reality TV show that promotes traditional recipes made with locally sourced and affordable ingredients. Complemented by a radio and social media campaign, PIFR and FAO also disseminated accessible information about the origin and nutritional composition of local foods, as well as the implications of dietary choices for human and planetary health. This form of food literacy and consumer education emphasizes the co-benefits of shifting to healthier diets, while supporting indigenous farmers and local economies. By reframing public narratives around food and nutrition, the programme contributed to building consumer agency and fostering transformative actions at all levels of society [11,29].

Additionally, partnerships with academia from the Pacific, New Zealand and Australia enabled the production of new data and comprehensive food systems assessments that identified inter-sectoral trends, systemic bottlenecks and critical trade-offs, thereby strengthening the science–policy interface. This evidence base enabled decision-makers to engage in dialogue around seemingly irreconcilable priorities, such as the trade-off between export-oriented, intensive cash crop farming and indigenous agroforestry and agroecology systems. Importantly, these assessments were not ends in themselves but inputs into inclusive, participatory governance processes. Multi-stakeholder platforms supported by the FIRST Programme convened a multiplicity of government agencies, the private sector and civil society to collectively explore policy options that combine both agro-industrial and agroecological approaches. This systems-based dialogue helped reframe competing priorities and balance economic, social and ecological benefits. These forums were essential to foster shared ownership for the formulation of Vanuatu’s Good Food Policy 2020−2030 and Fiji’s Policy on Food and Nutrition Security [11].

This strengthened science–policy interface also resulted in greater visibility of the role of rural women and youth in the country’s agrifood system. The Fiji Agriculture Census of 2020 incorporated age- and gender-disaggregated data for the first time and was accompanied by a gender analysis. These insights laid the groundwork for the government-led formulation of Fiji’s 2022−2027 Gender in Agriculture Policy, which seeks to promote structural change toward gender equality in agrifood systems at both institutional and community levels [30].

A defining feature of these processes has been the growing recognition that sustainable agrifood systems cannot be achieved without fully understanding and actively supporting the roles of stakeholders traditionally overlooked in top-down agricultural policymaking, particularly women and consumers. This marks a transformative reframing by valuing diverse forms of participation, shifting power dynamics and promoting more inclusive food systems. All these processes are the building blocks of a long-term journey towards more resilient Pacific islands food systems delivering healthy diets for all, while safeguarding natural resources and cultural integrity. The global agri-food systems community is now working with national governments to facilitate continuous investments in access to catalytic finance, essential for the implementation of these whole-of-government policy commitments. This includes the FAO’s commitment to develop the Pacific Small Island Developing States Investment Programme through a phased and flexible approach from 2025 to 2040 [11].

## 7. Rwanda: planning, learning and adaptation to reach ambitious goals

In the aftermath of years of civil war that plagued Rwanda and culminated in the genocide in 1994, the government set out on a path toward political stability and long-term development goals, aiming to achieve middle-income status by 2035 and high-income status by 2050. Central to this ambition has been the transformation of agrifood systems, given their critical relevance for the national economy and stability. Today, agriculture still employs around 56% of the population and contributes to around 27% of the country’s GDP [31,32]. Beyond economic concerns, the government recognized agrifood systems as strategic to addressing persistent food insecurity and malnutrition. Despite considerable reductions in poverty, stunting continues to impact nearly 30% of children [33], and prevalence of undernourishment still affects around one-third of the population [34]. Although socio-economic sustainability remains a priority, the past decade has seen growing focus on environmental sustainability, in particular in response to unsustainable land use and heightened climate vulnerability [35].

Recognizing that traditional approaches centred narrowly on agricultural production were insufficient, Rwanda progressively embraced a more integrated systems perspective to achieve food security and sustainable growth. To this end, over the past two decades, the government has gradually recalibrated its approach by enhancing inter-institutional coordination and creating space for more participatory policy processes [35,36]. This shift has enabled the establishment of agrifood systems governance mechanisms for policy planning, implementation and evaluation that encouraged the engagement of societal actors and the private sector. This approach is exemplified by the Agricultural Sector Working Group (ASWG), co-chaired by the Ministry of Agriculture and rotating development partners, and the Joint Action Development Forum at the district-level, which facilitates grassroots engagement, including from farmers’ organizations [35,37].

Since the UNFSS, the Rwandan Prime Minister’s Office has assumed a coordination role to align sectoral strategies and donor support with national objectives. In parallel, the government of Rwanda has translated its vision of shared prosperity into a coherent, unified national planning framework, ensuring all ministerial plans are aligned with overarching goals articulated in Vision 2020 [38] and the more recent Vision 2050 [39]. Within this framework, the agrifood systems is guided by the Strategic Plans for Agricultural Transformation (PSTA) formulated by the ASWG, now in its fifth iteration. The UNFSS process served as a catalyst for expanding the ambition of the PSTA, seeking to introduce cross-sectoral policy innovation. PSTA5, launched in December 2024, marks a more explicit commitment to a systems approach. By incorporating the UNFSS Pathways for Rwanda’s Food Systems Transformation, PSTA5 extends beyond food production and climate resilience to address interconnected outcomes such as gender equality, youth empowerment and rural–urban development.

Such an explicit transformation agenda has laid the groundwork for enhancing coherence among sectoral policies, budgets and outcomes. The Rwanda Environment Management Authority, supported by international development partners, played a pivotal role in mainstreaming environmental sustainability within agrifood policies. By generating compelling evidence of the economic benefits of more sustainable practices in increased agricultural productivity and cost savings, they influenced planning and resource allocation, particularly within the Ministry of Finance and Economic Planning. This resulted in further integration of environmental sustainability into the budgets of various policies and strategies, including the PSTAs [35].

In addition to inviting a diversity of interests and perspectives to influence decision-making and coherent resource allocation, as described above, the experience of Rwanda illustrates a transformative shift in its institutional commitment to systems thinking and adaptive learning. The government has fostered a culture of engaging in learning and reflection, through high-level leadership retreats focused on collective performance evaluation [36] and structured feedback mechanisms like Knowledge Seminars that assess lessons learned from previous PSTA cycles. The Policy Learning Programme launched in 2023, with support from FAO and national and international research institutes, aims at enhancing institutional agility and has contributed to the latest evolution of the PSTA. By equipping policymakers with systems thinking tools, Rwanda is building the capacity to manage complexity and steer transformative change [37,40].

## 8. Conclusions

The cases illustrated in this article show that some countries are gradually moving away from linear and siloed ways of thinking and working towards embracing elements of a systems approach, intentionally steering ongoing food systems transformations towards greater social, economic and environmental goals.

The authors acknowledge the limitations of this study. First, it is based on a limited number of cases; secondly, the information in the sources consulted was not aimed at explicitly collecting data on systems approaches. This partly explains why certain aspects related to a systems approach may be missing from the data and none of the examples encompasses all the elements of a systems approach presented at the beginning of this article. Notwithstanding these limits, certain reflections can be made concerning some aspects that stand out.

In most cases, changes were brought about over the course of several years of action and through iterations of planning cycles. It is the case of Costa Rica, for example, where the process of envisioning a transformation towards desirable outcomes started decades ago and is still in evolution. This indicates that agrifood systems do not transform overnight. The process of transformation in practice is more like a transition—the result of accumulated changes with transformational intent that eventually add up to shift the system to a transformed state. Transformation has a longitudinal dimension, and it will inevitably involve trial and error, as well as setbacks regarding desired achievements [22,27].

A second reflection concerns the role of leadership, governance arrangements and transformation catalysts. It was noted that to make a difference, a country’s leadership needs to show strong willingness and commitment to drive the transformation agenda and keep the momentum going [27], as in the cases of Morocco and Rwanda, where political leaders explicitly recognized the urgency of change and demonstrated re-orientation of policy and investments towards systems approaches, capitalizing on global processes around the UNFSS. At the same time, systems leaders must recognize the need for shared governance processes that mobilize multi-stakeholder, collective action [41] and break siloes of knowledge by engaging social actors in knowledge co-creation [42]. Noteworthy was the role of the FAO, and development partners in general, as brokers or facilitators of inclusive governance spaces that engage a diversity of actors in decision-making—as in the case of Albania—or generate new narratives—as in the case of the Pacific islands. By working to connect people and institutions to act more in concert with each other, enhance coherence among multidimensional outcomes of transformation, and amplify impacts across local, national and global scales, all the above entities have often acted as transformation catalysts [43,44], helping drive structurally transformative change.

A final reflection is that changing the direction of ongoing food systems transformation towards greater sustainability cannot be accomplished only through changes in policies, institutions and resources [11]. The authors observed a virtuous cycle of transformation, fostered by both tangible outputs (e.g. a policy or a governance body or an investment) and less tangible changes (e.g. in thinking, relationships, connections and agency), both essential for sustained impact [12]. For example, in the case of Albania, the dialogues led groups of local actors that had not previously worked together to build stronger connections, and in so doing to learn from one another, navigate differences and develop a common way of thinking. This empowered them to advocate for localized changes in national forums, leading to tangible results such as the creation of an agritourism department within MARD to improve joint cross-ministerial actions, which in turn further solidified the common vision. In Fiji, the tangible result of an agriculture policy that strongly includes gender considerations is the result of a series of science–policy platforms that allowed a heightened awareness of the gendered nature of the obesity pandemic in the island. This gave greater agency to certain groups—such as women—to have a voice in the process and to create stronger relationships with community-level leaders.

The above considerations lead to confirming what to some extent is already known about the implications of taking a systems approach. First, it is necessary to move beyond short-termism, with programmatic and investment cycles designed and implemented with a long-term vision. Secondly, the ecosystem of support to agrifood systems transformation needs to recognize and value the role of visionary leaders, by enhancing their capacity to leverage systems thinking; at the same time, adequate assistance needs to be provided for the creation of inclusive governance processes that build agency of underrepresented voices [45] and ensure a plurality of perspectives in the decision-making process [46].

The self-reinforcing link between tangible and intangible outputs of a systems approach has a dual implication; the first pertains to the importance of collecting information on both types of data, by developing monitoring, evaluation and learning (MEL) mechanisms tailored to systems change through participatory processes that capture also qualitative indicators of process, social capital and agency [47,48]. In so doing, MEL mechanisms would have to consider not only consolidated metrics of economic growth, such as GDP, but also more holistic metrics related to growth distribution and equity and other critical environmental and social outcomes of agri-food systems [49]. Second, there is a need to invest in agrifood systems interventions that consciously embrace and promote the core elements of a systems approach aiming at both tangible and intangible changes, as essential components of transformation.

These are preliminary reflections. Future lines of research would require comprehensive studies to analyse and document cases that illustrate the application of a systems approach in practice.

## Data Availability

This article has no additional data.

## Footnotes

^1^
The Food and Agriculture Organization of the United Nations (FAO) uses the term ‘agrifood systems’ to indicate the journey of food from farm to table. The systems also encompass non-food products that constitute livelihoods and all of the people, as well as the activities, investments and choices, that play a part in getting us these food and agricultural products. In the FAO Constitution, the term ‘agriculture’ and its derivatives include fisheries, marine products, forestry and primary forestry products [1].

## References

[1] FAO. 2021 Report of the Council of FAO – Hundred and Sixty-sixth Session: 26 April – 1 May 2021. Rome, CL 166/REP. Rome, Italy: Food and Agriculture Organization. See https://www.fao.org/3/nf693en/nf693en.pdf.

[2] United Nations. 2019 Independent group of scientists appointed by the Secretary-General. Global sustainable development report 2019: the future is now – science for achieving sustainable development. New York, NY: United Nations. See https://sdgs.un.org/sites/default/files/2020-07/24797GSDR_report_2019.pdf.

[3] FAO. 2022 The future of food and agriculture – drivers and triggers for transformation. Rome, Italy: Food and Agriculture Organization. (10.4060/cc0959en)

[4] UN Secretary-General. 2023 Making food systems work for people and planet. UN Food Systems Summit +2. See https://www.unfoodsystemshub.org/docs/unfoodsystemslibraries/stocktaking-moment/un-secretary-general/sgreport_en_rgb_updated_compressed.pdf?sfvrsn=560b6fa6_33.

[5] Zurayk R, Yehya AAK, Sadek M, De Matteis L, Valls Bedeau J. 2024 Pathways for agrifood systems transformation and regional cooperation in the Mediterranean. Rome, Italy: Food and Agriculture Organization. (10.4060/cd1714en)

[6] FAO. 2021 FAO strategic framework 2022–31. Rome, Italy: Food and Agriculture Organization. See https://openknowledge.fao.org/server/api/core/bitstreams/29404c26-c71d-4982-a899-77bdb2937eef/content.

[7] UN Sustainable Development Group (UNSDG). 2023 Six transitions: investment pathways to deliver the SDGs. New York, NY: United Nations. See https://sdgs.un.org/sites/default/files/2024-07/Six%20Transitions%20English.pdf.

[8] FAO. 2025 Transforming food and agriculture through a systems approach. Rome, Italy: Food and Agriculture Organization. (10.4060/cd6071en)

[9] Fazey I, Colvin J. 2023 Transformation: an introductory guide to fundamental change for researchers and change makers in a world of crises - a report for the Transforming UK Food Systems SPF Programme. York, UK: University of York/Stroud, UK: Emerald Network. See https://fixourfood.org/wp-content/uploads/2023/07/Transformation-introductory-guide-by-Fazey-and-Colvin.pdf.

[10] FAO, IFAD, WFP, UNICEF, WHO. 2021 The state of food security and nutrition in the world 2021. Transforming food systems for food security, improved nutrition and affordable healthy diets for all. Rome, Italy: Food and Agriculture Organization. (10.4060/cb4474en)

[11] FAO. 2024 Stories of agrifood systems change: insights from Côte d’Ivoire, Cambodia, the Pacific, Guatemala and Albania. Rome, Italy: Food and Agriculture Organization. (10.4060/cd1657en)

[12] Kania J, Kramer M, Senge P. 2018 The water of systems change. Boston, MA: Foundation Strategy Group. See https://www.fsg.org/wp-content/uploads/2021/08/The-Water-of-Systems-Change_rc.pdf.

[13] Woodhill J, Millican J. 2023 Systems thinking and practice: a guide to concepts, principles and tools for FCDO and partners. Brighton, UK: Institute of Development Studies. See https://hdl.handle.net/20.500.12413/17862.

[14] Dreier L, Nabarro D, Nelson J. 2019 Systems leadership for sustainable development: strategies for achieving systemic change. Cambridge, MA: Corporate Responsibility Initiative, Harvard Kennedy School. See https://www.hks.harvard.edu/sites/default/files/centers/mrcbg/files/Systems%20Leadership.pdf.

[15] Fazey I, Leicester G. 2022 Archetypes of system transition and transformation: six lessons for stewarding change. Energy Res. Social Sci. **91**, 102646. (10.1016/j.erss.2022.102646)

[16] McKee M, Fraj MB, Bergaoui K, Mohammed Y, McDonnell R. 2020 MENAdrought baseline assessment Morocco. Columbo, Sri Lanka: IWMI. See https://menadrought.iwmi.org/wp-content/uploads/sites/44/2020/08/BaselineMorocco_MENAdrought_final.pdf.

[17] Taheripour F, Tyner WE, Haqiqi I, Sajedinia E. 2020 Water scarcity in Morocco: analysis of key water challenges. Washington, DC: World Bank. (10.1596/33306)

[18] ONDH. 2021 Dynamiques des niveaux de vie et de la pauvreté au Maroc: une analyse longitudinale. Rabat, Morocco: Observatoire National du Développement Humain. See https://www.ondh.ma/sites/default/files/2021-11/Dynamique_pauvret%C3%A9_24%20nov%202021.pdf.

[19] Royaume du Maroc. 2021 M. Nasser Bourita: la sécurité alimentaire a toujours représenté une priorité stratégique pour le Maroc. In Ministère des Affaires étrangères de la Coopération Africaine et des Marocains résidant à l’étranger. Rabat, Morocco. See https://diplomatie.ma/fr/m-nasser-bourita-la-s%C3%A9curit%C3%A9-alimentaire-toujours-repr%C3%A9sent%C3%A9-une-priorit%C3%A9-strat%C3%A9gique-pour-le-maroc.

[20] Royaume du Maroc. 2023 Sécurité alimentaire : le Maroc accorde un intérêt particulier à la promotion du secteur agricole (M. Baitas). In Ministère de la Jeunesse, de la Culture, et de la Communication. Rabat, Morocco. See https://www.maroc.ma/fr/actualites/securite-alimentaire-le-maroc-accorde-un-interet-particulier-la-promotion-du-secteur.

[21] du Maroc R. 2024 SM le roi adresse un discours à la nation à l’occasion de la fête du trône. Rabat, Morocco: Chef du Gouvernement. See https://www.cg.gov.ma/fr/node/11814.

[22] FAO. 2024 Inter-country learning dialogue on multistakeholder governance for agrifood systems transformation. Rome, Italy: Food an Agriculture Organization. See https://www.fao.org/food-systems/news/news-detail/Inter-country-learning-dialogue-on-multistakeholder-governance-for-agrifood-systems-transformation/en (accessed October 23 October 2024).

[23] FAO. 2024 Let learning lead: a promising practice for agrifood systems transformation. emerging insights and innovations from the SFS-MED experience – report of the workshop "Agrifood Systems Learning for Change - the SFS-MED Experience", FAO Headquarters, 6 and 7 June 2024. Rome, Italy: FAO. See https://openknowledge.fao.org/handle/20.500.14283/cd2917en.

[24] United Nations Country Team, Albania. 2020 Common country analysis 2020. Tirana, Albania: United Nations Albania. See https://albania.un.org/sites/default/files/2021-02/Web_CCA_2020_final.pdf.

[25] Partalidou M, De Matteis L. 2024 Sustainable agritourism: an opportunity for agrifood systems transformation in the Mediterranean. Technical brief. Rome, Italy: Food and Agriculture Organization. See https://openknowledge.fao.org/handle/20.500.14283/cd3578en.

[26] FAO. 2024 Policymaking for agrifood systems transformation in Costa Rica. Rome, Italy: Food and Agriculture Organization. (10.4060/cc9866en)

[27] Guijt J, Wigboldus S, Brouwer H, Roosendaal L, Kelly S, Garcia-Campos P. 2021 National processes shaping food systems transformations – lessons from Costa Rica, Ireland and Rwanda. Rome, Italy: Food and Agriculture Organization. (10.4060/cb6149en)

[28] Roosendaal L, Brouwer H, Garcia Campos P, Prado-Rivera F. 2021 Costa Rica’s journey towards sustainable food systems – the processes and practices that made a difference. Rome, Italy: Food and Agriculture Organization. (10.4060/cb5997en)

[29] O’Meara L *et al*. 2023 Pacific food systems – the role of fish and other aquatic foods for nutrition and health. Apia, Samoa: Food and Agriculture Organization. (10.4060/cc5796en)

[30] FAO. 2023 Highlights of FAO’s programme in the Pacific islands. Apia, Samoa: Food and Agriculture Organization. (10.4060/cc4379en)

[31] World Bank. 2024 DataBank: World Development Indicators. Agriculture, forestry, and fishing, value added (% of GDP). Washington, DC: World Bank. See https://databank.worldbank.org/reports.aspx?source=2&series=NV.AGR.TOTL.ZS (accessed October 29 October 2024).

[32] World Bank. 2024 Employment in agriculture (% of total employment) (modeled ILO estimate). Washington, DC: World Bank. See https://data.worldbank.org/indicator/SL.AGR.EMPL.ZS?name_desc=true (accessed 29 October 2024).

[33] World Bank. 2024 DataBank: World Development Indicators. Prevalence of stunting, height for age (modeled estimate, % of children under 5). Washington, DC: World Bank. See https://databank.worldbank.org/reports.aspx?source=2&series=SH.STA.STNT.ME.ZS&country= (accessed October 29 October 2024).

[34] FAO. 2024 FAOSTAT: suite of food security indicators. Rome, Italy: Food and Agriculture Organization. See https://www.fao.org/faostat/en/#data/FS (accessed 30 October 2024).

[35] Wigboldus S, Guijt J, Garcia-Campos P. 2021 Rwanda’s journey towards sustainable food systems – the processes and practices that made a difference. Rome, Italy: Food and Agriculture Organization. (10.4060/cb6057en)

[36] Chemouni B. 2017 The politics of core public sector reform in Rwanda. Manchester, UK: Effective States and Inclusive Development Research Centre, University of Manchester. (10.2139/ssrn.3013650)

[37] FAO. In press Policymaking for agrifood systems transformation in rwanda. Rome, Italy: Food and Agriculture Organization.

[38] Republic of Rwanda. 2012 Rwanda Vision 2020 - Revised 2012. See https://climatechange.gov.rw/fileadmin/user_upload/Documents/Report/RwandaVision2020.pdf.

[39] Republic of Rwanda. 2020 Rwanda Vision 2050. Rwanda. See https://www.minaloc.gov.rw/fileadmin/user_upload/Minaloc/Publications/Useful_Documents/English-Vision_2050_full_version_WEB_Final.pdf.

[40] Ilie ET, Kelly S, Felici FB, Kattel R, Roll K. 2025 Workshop report on training for policymakers on agrifood systems transformation – in support of Rwanda’s fifth strategic plan for agriculture transformation. Rome, Italy: Food and Agriculture Organization. (10.4060/cd4341en)

[41] Leeuwis C, Boogaard BK, Atta-Krah K. 2021 How food systems change (or not): governance implications for system transformation processes. Food Security **13**, 761–780. (10.1007/s12571-021-01178-4) 34178184 PMC8211938

[42] IPES-Food. 2015 The new science of sustainable food systems. Overcoming barriers to food system reform. Brussels, Belgium: International Panel of Experts on Sustainable Food Systems. See https://www.ipes-food.org/_img/upload/files/NewScienceofSusFood.pdf.

[43] Lee JY, Waddock S. 2021 How transformation catalysts take catalytic action. Sustainability **13**, 9813. (10.3390/su13179813)

[44] Waddock S. 2022 Catalyzing purposeful transformation: the emergence of transformation catalysts. Business Soc. Rev. **127**, 167–170. (10.1111/basr.12263)

[45] Clapp J, Moseley WG, Burlingame B, Termine P. 2022 Viewpoint: the case for a six-dimensional food security framework. Food Policy **106**, 102164. (10.1016/j.foodpol.2021.102164)

[46] Duncan J *et al*. 2022 Democratic directionality for transformative food systems research. Nat. Food **3**, 183–186. (10.1038/s43016-022-00479-x) 37117640

[47] Estrella M, Blauert J, Campilan D, Gaventa J, Gonsalves J, Guijt IM, Johnson DA, Ricafort R. 2000 Learning from change: issues and experiences in participatory monitoring and evaluation. London, UK: Intermediate Technology Publications. See https://edepot.wur.nl/81366.

[48] Coger T, Corry S, Gregorowski R. 2021 Reshaping monitoring, evaluation, and learning for locally led adaptation. Washington, DC: World Resources Institute. (10.46830/wriwp.20.00060)

[49] Buckton SJ *et al*. 2024 Reform or transform? A spectrum of stances towards the economic status quo within ‘new economics.’ Discours. Glob. Social Challenges J. **3**, 382–421. (10.1332/27523349Y2024D000000025)

